# Lowering barometric pressure induces neuronal activation in the superior vestibular nucleus in mice

**DOI:** 10.1371/journal.pone.0211297

**Published:** 2019-01-25

**Authors:** Jun Sato, Hideaki Inagaki, Mayu Kusui, Makoto Yokosuka, Takahiro Ushida

**Affiliations:** 1 Department of Physical Therapy, College of Life and Health Sciences, Chubu University, Kasugai-shi, Aichi, Japan; 2 Multidisciplinary Pain Center, Aichi Medical University, Nagakute-shi, Aichi, Japan; 3 Faculty of Veterinary Science, School of Veterinary Medicine, Nippon Veterinary and Life Science University, Musashino-shi, Tokyo, Japan; Central South University, The Third Xiang Ya Hospital, CHINA

## Abstract

Weather changes accompanied by decreases in barometric pressure are suggested to trigger meteoropathy, i.e., weather-related pain. We previously reported that neuropathic pain-related behavior in rats is aggravated by lowering barometric pressure, and that this effect is abolished by inner ear lesions. These results suggest that mechanisms that increase vestibular neuronal activity may parallel those that contribute to meteoropathy generation. However, it remains unknown whether changes in barometric pressure activate vestibular neuronal activity. To address this issue, we used expression of c-Fos protein as a marker for neural activation. Male and female mice were placed in a climatic chamber, and the barometric pressure was lowered by 40 hPa, from 1013 hPa, for 50 min (LP stimulation). The total number of c-Fos-positive cells in the vestibular nuclei was counted bilaterally after LP stimulation. We also video-recorded mouse behaviors and calculated the total activity score during the LP stimulation. LP stimulation resulted in significant c-Fos expression in the superior vestibular nucleus (SuVe) of male and female mice. There was no effect of LP stimulation on the total activity score. These data show that distinct neurons in the SuVe respond to LP stimulation. Similar mechanisms may contribute to the generation of meteoropathy in humans.

## Introduction

It has long been assumed that weather changes can trigger episodes of meteoropathy, such as headache and other forms of chronic pain [1–6]. Meteorological factors that influence pain include barometric pressure, humidity, wind, precipitation, and temperature [6–9]. We have previously demonstrated that lowering barometric pressure (5–27 hPa lower than atmospheric pressure; LP stimulation) using a climatic chamber leads to increased pain-related behaviors in rats with chronic constriction injury (CCI) [10] and monoarthritic rats [11]. We have also reported that the LP-induced increase in pain-related behaviors vanishes after drug-induced destruction of the inner ear in CCI rats [12]. In another study, we extracellularly recorded neural activities in vestibular nuclei with a glass microelectrode and examined the effect of LP (40 hPa/ 8 min) in normal anesthetized rats. Seven out of 20 recorded vestibular neurons increased their discharge frequency upon LP stimulation [13]. These results suggest that the barometric sensor/sensing system influencing nociceptive behavior during LP in CCI rats is located in the inner ear. However, it is not known whether changes in barometric pressure activate vestibular neuronal activity in mice. If so, mechanisms that increase vestibular neuronal activity may parallel those that contribute to the development of meteoropathy. To investigate this issue, in this study, we used the expression of the immediate early-gene c-Fos, as a marker for neuronal activity in the vestibular nuclei and found that distinct vestibular neurons indeed respond to LP stimulation.

## Materials and methods

### Animals

Male (n = 18) and female (n = 16) C57BL/6J mice (14-weeks-old at the beginning of the experiments) were used in this study (Charles River Laboratories Japan, Kanagawa, Japan). The mice were housed in plastic cages and kept in a regulated environment (23 ± 1°C; 50 ± 5% relative humidity) with a 12-h light-dark cycle (lights on at 08:00). Food (Oriental MF; Oriental Yeast Co., Tokyo, Japan) and tap water were available ad libitum. All experiments were performed in accordance with the Guidelines for Animal Experiments of Chubu University and Aichi Medical University, and the United States National Institutes of Health Guide for the Care and Use of Laboratory Animals. All protocols for animal experiments were approved by the Animal Experiment Committee of Chubu University (No. 3010074) and Aichi Medical University (No. 2018–43).

### Lowering of barometric pressure

In the present experiment, we used a pressure-controlled climatic chamber, which is able to lower barometric pressure at a variety of rates and ranges [14]. The chamber used can maintain its own barometric pressure independently of the atmospheric pressure changes outside.

**Fig 1:**
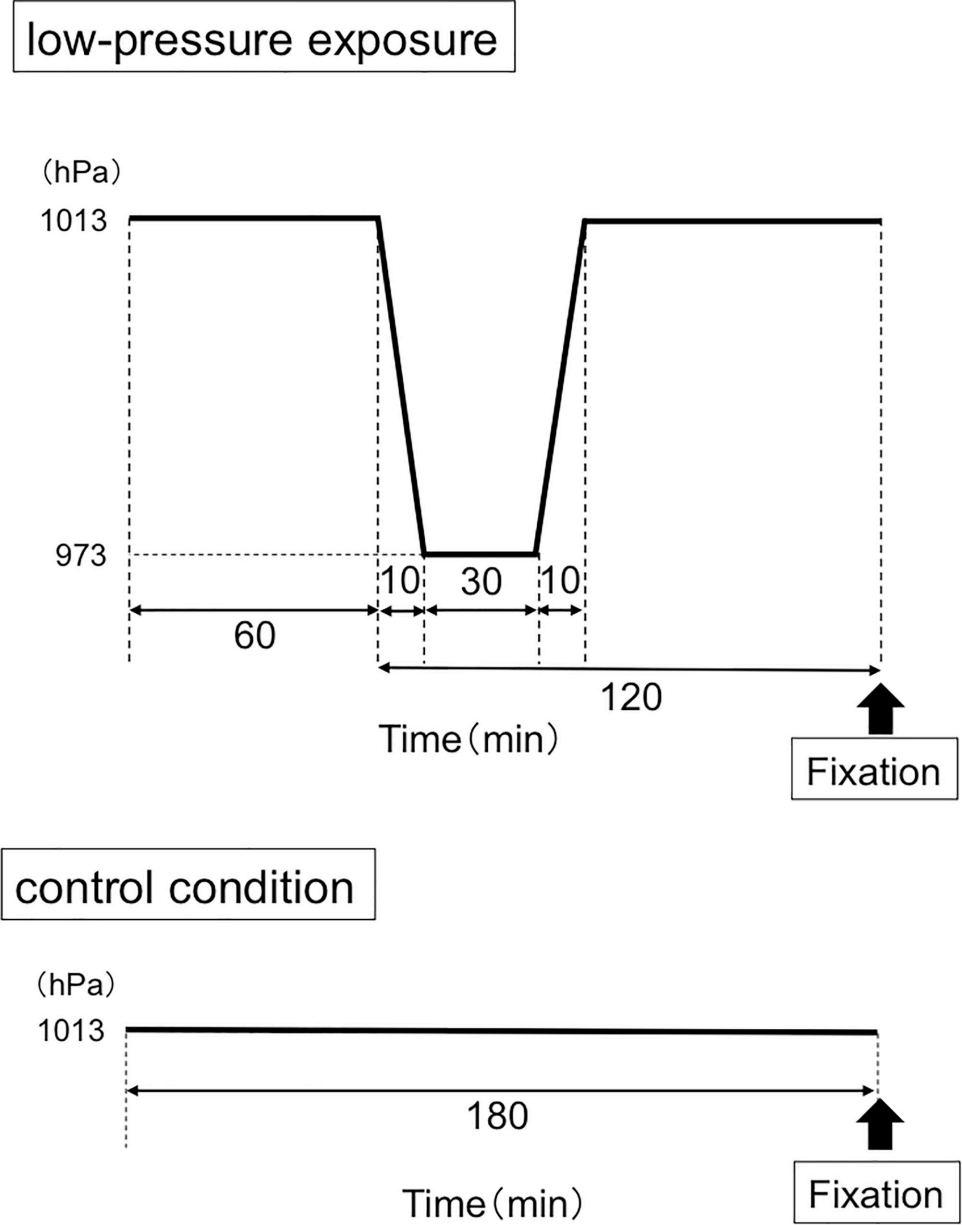
**Time schedule of low-pressure exposure (upper panel) and control condition (lower panel).** Barometric pressure was lowered from 1013 hPa to 973 hPa.

Before the experiments, animals were acclimated for 60 min in the chamber (barometric pressure: 1013 hPa, ambient temperature: 22 ± 2°C, relative humidity: 50 ± 10%) on 2 consecutive days. On the experimental day, mice were placed in the chamber set at the basal barometric pressure (1013 hPa) for 60 min (ambient temperature: 22 ± 2°C, relative humidity: 50 ± 10%). The barometric pressure was then lowered by 40 hPa over the course of 10 min, kept at this level for 30 min, and then returned to the normal level over the course of 10 min (LP stimulation). After returning to the basal pressure level (1013 hPa), mice were placed in the chamber for 70 min (Fig 1). A group of animals was placed in the chamber at 1013 hPa without pressure changes for 180 min and served as the control group.

### Animal activity

We tested the effect of LP stimulation on the activity of animals. The behavior of each mouse during the LP stimulation was recorded using a camera (Webcam C500; Logicool, Tokyo, Japan). As the total activity score, we calculated the total time that each animal spent walking, rearing, sniffing, and grooming during the 30 min before and 30 min after the beginning of LP stimulation. In control animals, the total activity score was calculated over a period of 60 min without LP stimulation. A researcher who was blind to the experimental conditions analyzed the behavior of the mice.

### Animal treatment and tissue preparation

After LP stimulation or 180-min observation (control), mice were deeply anesthetized with sodium pentobarbital, and perfused transcardially with saline, followed by 4% paraformaldehyde in 0.1 M phosphate-buffered saline (pH 7.4). The brains were removed, post-fixed overnight in the same fixative solution at 4°C, and then placed in 30% sucrose in 0.1 M phosphate-buffered saline (pH 7.4) for cryoprotection.

### c-Fos immunocytochemistry

Serial coronal sections (40-μm thick) of each brain were cut on a cryostat. Every two sections that contained the vestibular nuclei were used for immunocytochemistry to detect c-Fos protein (17–22 sections/mouse), and the remaining sections were stained with cresyl violet (1% in water). Sections for immunocytochemistry were rinsed with 0.1 M phosphate-buffered saline (PBS, pH 7.4) and then treated with 3% hydrogen peroxide in PBS for 15 min. They were then washed with PBS for 20 min with one change and rinsed with 0.3% Triton X-100 in 0.1 M phosphate buffer (PBST, pH 7.4) for 20 min with one change; nonspecific binding sites were blocked by incubation in 25% Block Ace (DS Pharma Biomedical, Osaka, Japan) in PBST for 20 min at room temperature. The sections were subsequently incubated with an anti-human c-Fos antibody [rabbit monoclonal IgG: c-Fos (9F6) Rabbit mAb; Cell Signaling Technology, Beverly, MA, USA] diluted 10,000 times with 10% Block Ace in PBST for approximately 40 h at 4°C. After three 10-min washes with PBST, the sections were incubated with a biotinylated anti-rabbit secondary antibody (BA-1000; Vector Laboratories, Burlingame, CA, USA) diluted 500 times with PBST for 2 h at room temperature, and then processed with the ABC kit (VECTASTAIN Elite ABC kit PK-6100; Vector Laboratories) appropriately diluted with PBST. Each step was followed by three 10-min washes with PBST. After the last wash, the sections were immersed in 0.175 M sodium acetate buffer (pH 7.4) for 30 min with two changes, and then appropriately incubated with the chromogen solution [0.02% 3,3'-diaminobenzidine, 0.0025% hydrogen peroxide, and 0.25% nickel (II) chloride hexahydrate in 0.175 M sodium acetate buffer]. The reaction was stopped by transferring the sections to 0.175 M sodium acetate buffer, and finally washing them with PBS for 20 min, with one change, and mounting them on gelatin-coated glass slides.

### Quantification of c-Fos immunoreactivity

The total number of c-Fos-immunopositive cells in the superior (SuVe), lateral (LVe), medial (MVe), and spinal (SpVe) vestibular nuclei was counted by an experimenter blind to the experimental conditions. c-Fos-positive cells were counted in each nucleus under a microscope at a magnification of 100×, with results expressed as the total number of c-Fos-positive cells in each nucleus, bilaterally. Each vestibular nucleus was identified by cresyl violet staining of sections adjacent to those used for c-Fos immunostaining, according to a mouse brain atlas [15].

### Data analyses

Data are displayed as the mean ± standard error (SE). Statistical analyses were performed using a two-way analysis of variance (ANOVA) followed by a post hoc Tukey-Kramer test to determine the effect of the barometric pressure condition and sex on the number of c-Fos-immunoreactive cells in each vestibular nucleus and on the total activity scores. The criterion for statistical significance was set at p < 0.05 for all comparisons.

## Results

**Fig 2:**
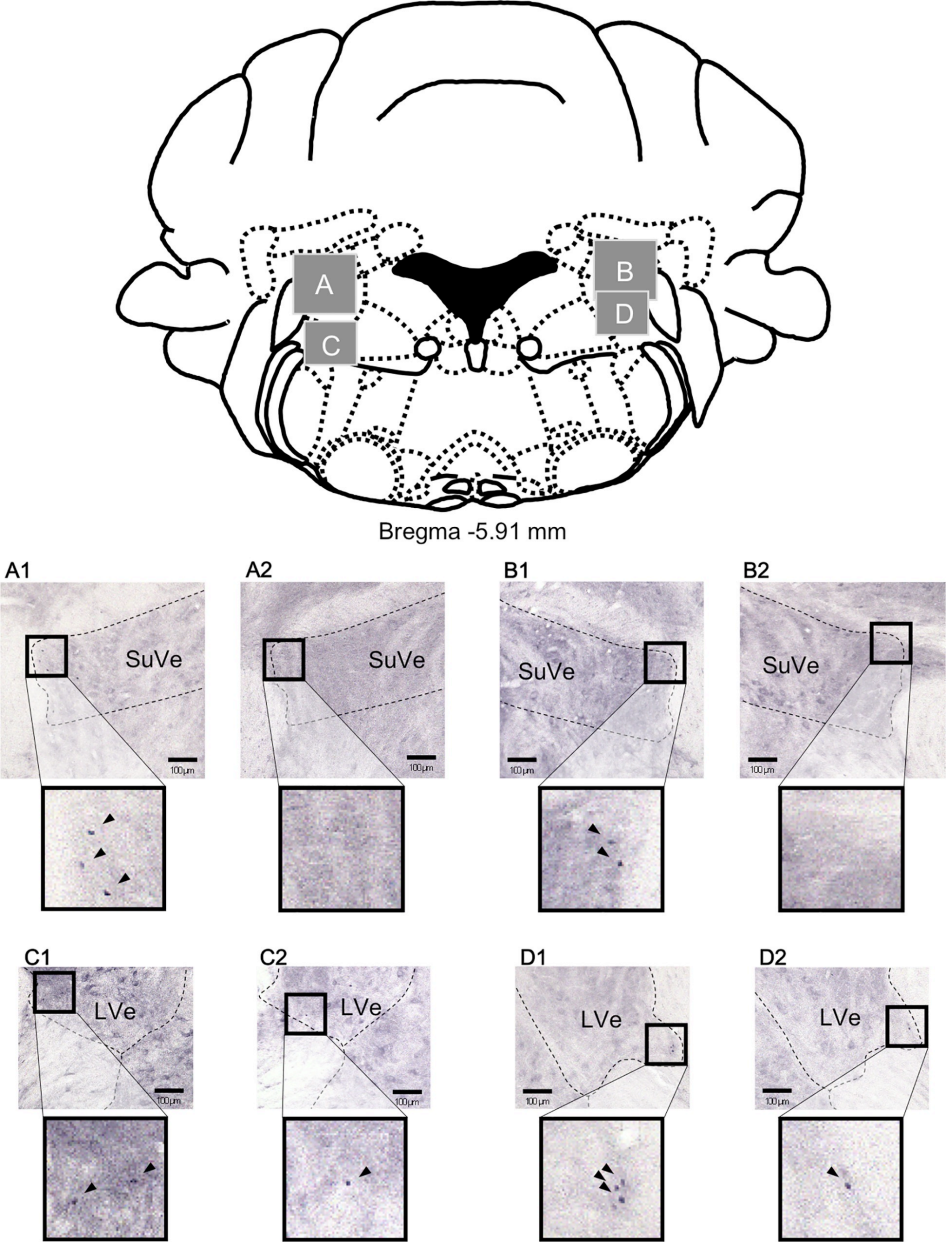
**Photomicrographs of the superior vestibular nucleus (SuVe) (A: left and B: right) and the lateral vestibular nucleus (LVe) (C: left and D: right) in male mice 2 h after low-pressure exposure (A1, B1, C1, and D1) and under control conditions (A2, B2, C2, and D2).** Arrowheads indicate c-Fos-positive cells. The histological sections are shown as gray squares in the schematic drawing of the medulla oblongata (upper panel).

**Fig 3:**
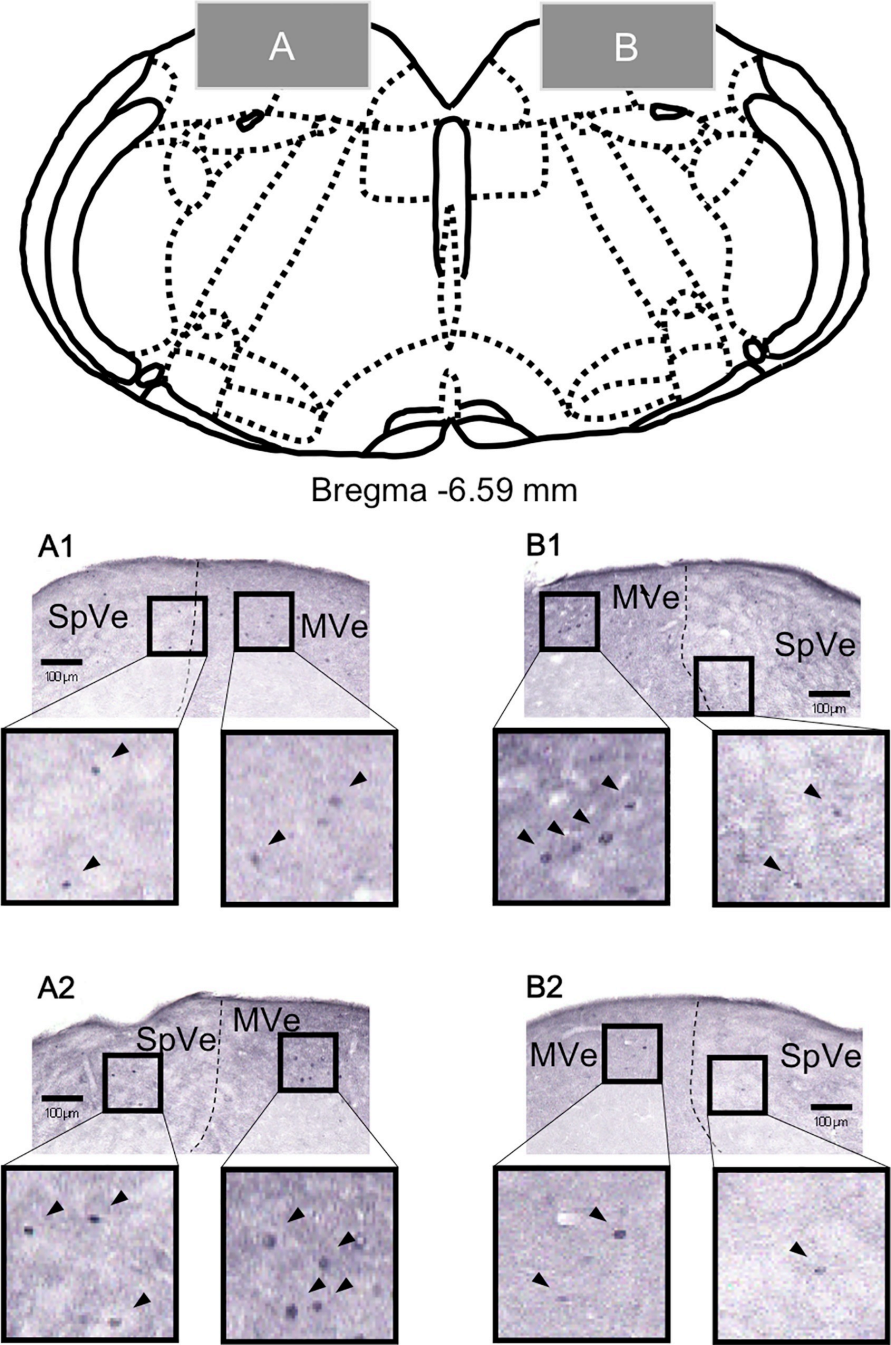
**Photomicrographs of the spinal vestibular nucleus (SpVe) and medial vestibular nucleus (MVe) (A: left and B: right) in male mice 2 h after low-pressure exposure (A1 and B1) and under control conditions (A2 and B2).** Arrowheads indicate c-Fos-positive cells. The histological sections are shown as gray squares in the schematic drawing of the medulla oblongata (upper panel).

**Fig 4:**
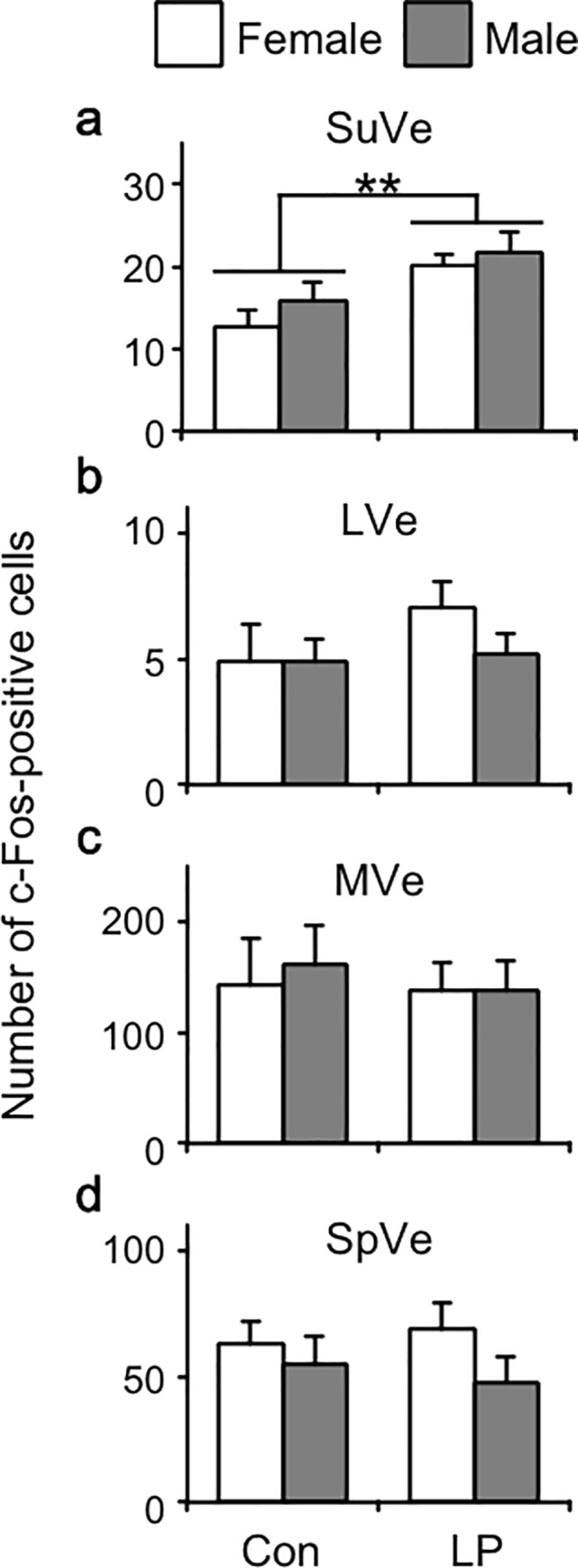
Number of c-Fos-positive cells in each vestibular nucleus. Quantification of c-Fos-positive cells in the (a) superior vestibular nucleus (SuVe), (b) lateral vestibular nucleus (LVe), (c) medial vestibular nucleus (MVe), (d) spinal vestibular nucleus (SpVe), in female (*n* = 8 per group) and male (*n* = 9 per group) mice 2 h after lowering barometric pressure stimulation (LP: 973 hPa) or under control conditions (Con: 1013 hPa). Each bar represents the mean + standard error; **, *p* < 0.01 (two-way ANOVA followed by Tukey-Kramer test).

We examined whether LP stimulation induced neural activation in the vestibular nuclei. For this analysis, vestibular nuclear segments were immunohistologically examined for c-Fos protein, a marker of neuronal activation. The photomicrographs in Fig 2 were taken from SuVe and LVe sections bilaterally (SuVe: A and B, LVe: C and D) from male mice exposed to the LP stimulation (A1, B1, C1, and D1) or to control conditions (A2, B2, C2, and D2). The photomicrographs in Fig 3 were taken from SpVe and MVe sections bilaterally (A and B) from male mice exposed to the LP stimulation (A1 and B1) or to control conditions (A2 and B2). The tissues were processed for c-Fos immunostaining. As shown in Fig 2, we found c-Fos immunoreactivity in some SuVe cells after LP stimulation (A1 and B1), but little or no immunoreactivity was observed under control conditions (A2 and B2). Fig 4 shows the average number of c-Fos-immunopositive cells for each vestibular nucleus. This number was significantly increased in the SuVe of mice of both sexes exposed to LP stimulation. Two-way ANOVA revealed a significant effect of barometric pressure (F1, 30 = 9.76, p < 0.01) but not sex, and no significant interaction between the two factors. Post hoc analysis indicated that LP stimulation significantly increased the average number of c-Fos-positive cells in the SuVe (p < 0.01). In other vestibular nuclei, namely in the LVe, MVe, and SpVe, the number of c-Fos-immunopositive cells in each nucleus was not affected by barometric pressure or sex (two-way ANOVA, p > 0.05).

Table 1 shows the mean total activity scores for female and male mice over a period of 60 min in the control condition (without LP stimulation) and those during the 30 min before and 30 min after the beginning of LP stimulation. In the control condition, the mean total activity scores of male mice were relatively larger than those of female mice, but the difference was not significant (p = 0.06). Moreover, there were no significant effects of LP stimulation and sex on the total activity scores.

**Table 1:** Total activity scores for female and male mice. Behaviors of each mouse during the 30 min before and 30 min after the beginning of LP stimulation were recorded (LP exposure). In control animals, the total activity score was calculated over a period of 60 min without LP stimulation (Control condition). Each number represents the mean ± standard error of the activity score.

	Control condition	LP exposure
**Female (*n* = 8 per group)**	361 ± 58.3	383 ± 106
**Male (*n* = 9 per group)**	761 ± 333	608 ± 131

## Discussion

This is the first study investigating the impact of barometric pressure changes on neuronal activity in the vestibular nuclei in mice. The rationale of the present study was to examine if changes in barometric pressure within a range of natural weather changes may influence the activity of second-order neurons in the vestibular nuclei of mice, particularly of neurons receiving vestibular afferent input. Our present data clearly demonstrate that distinct neurons in the SuVe respond to LP stimulation. These data are consistent with our previous observation that some vestibular neurons in rats increased their discharge frequency upon LP stimulation [13].

Nishihara et al. reported that the air volume in the middle ear cavity is important for the transmission of slowly changing atmospheric pressure to the perilymph in the inner ear [16]. It has also been reported that pressure changes in the middle ear cavity induce a pressure difference between the perilymph and endolymph, and that this difference causes an increase in vestibular neuronal activity [17–19]. These results, although obtained using a much larger and more rapid pressure change (200 mm H_2_O or -200 mm H_2_O within 8.5 s) than the one used in the present study, suggest that LP stimulation induces a relative overpressure in the middle ear cavity and, thus, results in vestibular nuclei activation. This hypothesis is supported by the fact that the activity levels of laboratory mice change along with natural barometric pressure fluctuations [20], indicating that mice can sense such changes. Taken together, our present data suggest that a barometric sensor or sensing system that detects atmospheric pressure is present in the inner ear of mice.

It has long been known that barometric pressure-sensing abilities may be common in wild vertebrates, especially ones that are small in size, for whom even individual storms can be life-threatening events. For example, birds have been reported to sense and respond to decreases in barometric pressure [21]. In addition, sensitivity to small barometric pressure changes as low as 1–2 hPa has also been reported in these animals [22]. Birds probably detect barometric pressure using the paratympanic organ (PTO), located in the middle ear, which is mechanoreceptive [23]. The PTO is thought to function as both a barometer and an altimeter, helping birds to detect changes in both weather and altitude during migration. In birds, the PTO is innervated by the facial nerve, projecting to the vestibular nuclei [24] and may mediate barometric perception [25]. In humans, no comparable system for sensing small barometric pressure changes is presently known. However, rapid and large pressure changes during diving or flight have occasionally been found to induce transient and reversible vertigo (alternobaric vertigo) [26,27]. Persistent alternobaric vertigo has also been reported in patients with nasal obstruction and obstructive sleep apnea at ground level [28,29]. We have previously reported that weather-sensitive patients suffering from pain show lowered thresholds for self-motion perception in response to galvanic vestibular stimulation [30].

The present findings show that LP stimulation increases c-Fos immunoreactivity in the SuVe but not in the other vestibular nuclei, with no significant differences between male and female mice. It is known that neurons in the SuVe receive input principally from the ampullae of the semicircular ducts [31–33] suggesting that a barometric sensor or sensing system might be located in the semicircular ducts in mice of both sexes. Further studies are needed in order to clarify the pressure sensing mechanism underlying this system.

How can vestibular neural activities be triggering factors for episodes of meteoropathy? It is possible that the influence of vestibular activation on autonomic functions, which occurs through modification of autonomic centers in the brain stem, also affects sympathetic nerve activities [34,35]. We have previously shown that LP increases blood pressure and heart rates of rats and mice [36] and in weather-sensitive patients [30]. These results indicate that changes in barometric pressure induce sympathetic activation both in rodents and humans. The influence of sympathetic nerve activities on nociceptive afferents after nerve injury is well documented [37–39]. In addition, we previously showed that sympathectomy inhibits LP-induced aggravation of mechanical hyperalgesia in nerve-injured rats, suggesting the involvement of sympathetic nerve activity in this phenomenon [10]. Based on these previous reports, we can assume that vestibular neuronal activation induced by LP stimulation increases pain through sympathetic nerve activities. Another possibility is that vestibular neural activities induce hormonal changes. This hypothesis is based on studies reporting that vestibular nuclear neurons in rats and humans project to the hypothalamus [40] [41], thus possibly modulating the hypothalamic-pituitary-adrenal axis (HPA-axis), one of the main bodily stress systems. It is well known that several chronic-pain conditions are related to the HPA-axis [42–44] and, thus, we can assume that hormones are released from the adrenal cortex in response to LP stimulation. These circulating hormones might directly activate the peripheral nociceptive fibers and induce vasoconstriction, thus increasing pain. Moreover, cutaneous nociceptive fibers have been reported to become responsive to adrenaline and noradrenaline after nerve injury [37,39,45].

In conclusion, the present study shows that distinct neurons in the SuVe respond to changes in barometric pressure. Similar mechanisms may contribute to the development of meteoropathy in humans, induced by changes in barometric pressure. How end organs in the inner ear sense barometric pressure changes remains to be elucidated.

## Supporting information

S1 Table All data for Fig 4. (PDF)

S2 Table All data for Table 1. (PDF)
