# Supplementary material for: Lowering barometric pressure induces neuronal activation in the superior vestibular nucleus in mice
Source: PLoS One. 2019 Jan 25;14(1):e0211297. doi: 10.1371/journal.pone.0211297 (PMC6347159; doi:10.1371/journal.pone.0211297)
Supplement: S1 Table — (PDF) [file pone.0211297.s001.pdf]

S1 Table

female					male				
mouse No.	SuVe	LVe	MVe	SpVe	mouse No.	SuVe	LVe	MVe	SpVe
control 1	14	7	28	58	control 1	12	6	260	84
control 2	9	14	82	108	control 2	20	9	372	110
control 3	6	1	131	63	control 3	18	2	154	35
control 4	5	2	49	83	control 4	12	7	36	24
control 5	14	4	93	65	control 5	8	3	188	26
control 6	22	5	218	39	control 6	5	3	24	14
control 7	13	2	147	22	control 7	20	4	139	51
control 8	19	4	397	66	control 8	24	8	153	89
low pressure 1	18	12	54	82	control 9	23	2	122	61
low pressure 2	25	6	176	120	low pressure 1	15	5	112	35
low pressure 3	23	3	116	89	low pressure 2	14	7	99	87
low pressure 4	16	7	46	70	low pressure 3	18	3	33	15
low pressure 5	20	5	224	45	low pressure 4	30	3	191	33
low pressure 6	21	5	116	45	low pressure 5	21	5	111	17
low pressure 7	23	11	229	30	low pressure 6	14	4	60	23
low pressure 8	16	7	188	47	low pressure 7	36	7	309	99
					low pressure 8	25	10	128	54
					low pressure 9	22	3	196	66
