# Supplementary material for: Lowering barometric pressure induces neuronal activation in the superior vestibular nucleus in mice
Source: PLoS One. 2019 Jan 25;14(1):e0211297. doi: 10.1371/journal.pone.0211297 (PMC6347159; doi:10.1371/journal.pone.0211297)
Supplement: S2 Table — (PDF) [file pone.0211297.s002.pdf]

S2 Table

	control	low pressure
Female	452.671875	994.453125
	371.484375	160.328125
	702.109375	139.15625
	184.703125	114.65625
	379.953125	159.34375
	205.6875	456.25
	1060.75	139.03125
	214.3554688	508.296875
Male	358.890625	721.1992188
	1276.894531	73.26953125
	1059.160156	509.4960938
	558.3007813	1035.023438
	109.5	1658.839844
	537.296875	1036.242188
	523.671875	591.4335938
	3274.898438	351.75
	90.50390625	415.421875
